# Draft Genome Sequences of Four Salmonella enterica subsp. enterica Serovar Gallinarum Strains Isolated from Layer Breeder Flocks in an Outbreak of Fowl Typhoid in Colombia

**DOI:** 10.1128/MRA.00122-19

**Published:** 2019-04-25

**Authors:** Ruy D. Chacón, Quézia Moura, Claudete S. Astolfi-Ferreira, David I. De la Torre, Laura M. C. Guerrero, Luz A. R. Martínez, Alexandra N. Corzo, Ney Hernando Morales López, Manuel Ramírez, Nilton Lincopan, Antonio J. Piantino Ferreira

**Affiliations:** a Department of Pathology, School of Veterinary Medicine, University of São Paulo, São Paulo, Brazil; b Department of Microbiology, Institute of Biomedical Sciences, University of São Paulo, São Paulo, Brazil; c Laboratory of Bacterial Zoonosis, National Institute of Health, Lima, Peru; University of Maryland School of Medicine

## Abstract

This report describes the genome sequences of four Salmonella enterica subsp. enterica serovar Gallinarum (*Salmonella* Gallinarum) strains isolated in Colombia in 2017 from layer breeders of different ages. The layer breeder flocks were presenting with an elevated mortality with lesions typical of fowl typhoid (FT). These draft genome sequences revealed a highly conserved genome of *Salmonella* Gallinarum strains circulating in Colombia.

## Abstract (precis)

This report describes the genome sequences of four Salmonella enterica subsp. enterica serovar Gallinarum (*Salmonella* Gallinarum) strains isolated in Colombia in 2017 from layer breeders of different ages. The layer breeder flocks were presenting with an elevated mortality with lesions typical of fowl typhoid (FT).

## ANNOUNCEMENT

Salmonella enterica subsp. enterica serovar Gallinarum (*Salmonella* Gallinarum) is the etiological agent of fowl typhoid (FT) in poultry, affecting mainly layer hens close to the egg-laying phase (1). *Salmonella* Gallinarum is responsible for elevated economic losses in different Latin American (LA) countries (2, 3). This pathogen can be detected in progeny and in adult birds, although it is most common in adults (4). However, in many cases, the bacteria can persist in tissues, which explains outbreaks in adult chickens that have not had previous contact with a reservoir (1).

This report describes the genome sequences of four *Salmonella* Gallinarum strains isolated in Colombia in 2017 from layer breeders of different ages, including those 13 weeks old (isolate GDX7), 25 weeks old (isolate GDX58), 15 weeks old (isolate FTA303), and 1 week old (isolate Buga). Each production unit of layer breeder flocks was composed of 10,000 chickens, and mortality reached 40,000 chickens per week, for a total of 500,000 birds. Some moribund birds were sacrificed and subjected to necropsy examination and presented with liver and spleen enlargement, necrotic foci, and friability. Liver and spleen fragments were collected and cultured in tetrathionate for 48 h at 37°C and then cultured in MacConkey and xylose-lysine-tergitol 4 (XLT4) agar for 24 h at 37°C. Typical colonies were detected in both medium types and were subjected to biochemical testing using the API 20E kit (bioMérieux SA, France). After biochemical identification, colonies were cultured in LB broth for 18 hours at 37°C for DNA extraction, using the ChargeSwitch genomic DNA (gDNA) mini bacteria kit (Thermo Fisher Scientific, Carlsbad, CA, USA). DNA concentration was determined by Qubit fluorometric quantitation and the double-stranded DNA (dsDNA) broad-range (BR) assay kit (Thermo Fisher Scientific) before being diluted to 0.5 ng/µl. A library was prepared using the Nextera XT DNA library preparation kit (Illumina, Inc., San Diego, CA, USA), and sequencing was performed using the MiSeq sequencing system (Illumina, Inc.), with a v2 kit, 500 cycles of amplification, and paired-end sequencing (2 × 250-bp reads). The *de novo* genome assembly of reads, adapter removal, and quality filtering were conducted using A5-miseq software version 20160825 (5). Additionally, the CAP3 software was used for a second assembly (6), and the sequences were annotated with the NCBI Prokaryotic Genome Annotation Pipeline (PGAP) version 4.6 (7). The total number of paired-end reads ranged from 1,292,434 to 3,004,038, with fragments of 205 to 305 bp, and the contigs used for assembly were >608 bp. The rest of the genomic and statistic information obtained in this study is summarized in Table 1.

**TABLE 1:** Genome sequencing results of *Salmonella* Gallinarum strains isolated from layer breeders in Colombia^*a*^

Strain name	No. of CDSs	No. of ncRNAs	No. of tRNAs	No. of functional proteins	G+C content (%)	No. of contigs	Contig size (bp)	No. of paired-end reads	Total length (bp)	*N*_50_ value	Genome coverage (×)	GenBank accession no.
GDX7	4,777	11	69	4,359	52.2	52	608–1,297,497	1,557,168	4,727,304	406,213	∼77.0	QYTY00000000
GDX58	4,777	11	68	4,366	52.2	43	619–1,297,593	1,668,814	4,728,049	511,172	∼83.0	QYTX00000000
FTA303	4,800	11	69	4,394	52.2	45	608–401,267	3,004,038	4,761,311	281,779	∼150.0	QZND00000000
Buga	4,718	11	69	4,325	52.2	26	945–1,046,337	1,292,434	4,711,572	401,648	∼64.0	RHEL00000000

a CDSs, coding DNA sequences; ncRNAs, noncoding RNAs.

Bacterial typing was performed with the *Salmonella* In Silico Typing Resource (SISTR) (8) and showed a multilocus sequence type (MLST) corresponding to sequence type 78 (ST-78) for all *Salmonella* isolates, which is common for *Salmonella* Gallinarum. Plasmid detection performed with PlasmidFinder version 2.0 (9) identified IncFII(S) in all isolates. Bacterial *in silico* phenotyping performed with ResFinder version 3.1 (10) identified the *aac(6')-Iaa* gene in all isolates, which is a chromosomally encoded aminoglycoside acetyltransferase that confers aminoglycosides. Outbreaks of FT in LA are recurrent due to several factors, such as chicken breeder suppliers, environmental contamination, installations, insects, wild bird reservoirs, hematophagous mites, and others. A genome comparison of different isolates from Colombia showed that the isolates are very similar. The currently described genomes of *Salmonella* Gallinarum isolates from layer breeder flocks can provide more information on *Salmonella* Gallinarum genetic evolution and genome organization and its pathobiological relationship with chickens for understanding certain outbreaks (11).

### Data availability.

These annotated draft genome sequences have been recorded in DDBJ/EMBL/GenBank under accession numbers QYTY00000000 (GDX7), QYTX00000000 (GDX58), QZND00000000 (FTA303), and RHEL00000000 (Buga) and Sequence Read Archive (SRA) accession numbers SRR8521149 (GDX7), SRR8521221 (GDX58), SRR8521219 (Buga), and SRR8521220 (FTA303).
